# Mechanical Analysis of Hybrid Polymeric Composites Reinforced with Recycled Eucalyptus and Montmorillonite Clay

**DOI:** 10.3390/polym18040445

**Published:** 2026-02-10

**Authors:** Juam Carlos Pierott Cabral, Victor Paes Dias Gonçalves, Michel Oliveira Picanço, Carlos Maurício Fontes Vieira, Noan Tonini Simonassi, Felipe Perisse Duarte Lopes

**Affiliations:** 1 Advanced Materials Laboratory, State University of the North of Rio de Janeiro Darcy Ribeiro—UENF, Av Alberto Lamego, 2000, Campos dos Goytacazes 28013-602, RJ, Brazil; juampierott.arq@gmail.com (J.C.P.C.); perisse@uenf.br (F.P.D.L.); 2 Agricultural Sciences and Engineering Center, Federal University of Espírito Santo, Av. Governador Lindemberg, 316, Jerônimo Monteiro 29550-000, ES, Brazil

**Keywords:** composites, eucalyptus powder, epoxy, montmorillonite clay, hybrid

## Abstract

Recent advances in polymeric composites emphasize the incorporation of natural and mineral fillers to enhance sustainability while maintaining mechanical performance. Studies have shown that lignocellulosic residues and nanostructured clays can improve stiffness and thermal stability, although interfacial compatibility remains a key challenge. This study investigates the mechanical behavior of epoxy composites reinforced with eucalyptus powder and montmorillonite clay, aiming to develop sustainable materials with reduced environmental impact. Formulations containing 5%, 10%, and 20% by volume of each particulate, as well as hybrid combinations, were produced and tested for impact, flexural, and compressive strength. Higher particulate contents were not explored, as fractions above 20% considerably increased viscosity, hindering proper mixing and specimen fabrication. Scanning electron microscopy (SEM) revealed irregular morphologies and heterogeneous dispersion of both fillers. The reduction in impact strength observed across all formulations was mainly attributed to poor interfacial adhesion and void formation, as no chemical or surface treatments were applied to enhance compatibility between the particulates and the epoxy matrix. Conversely, compressive strength improved at low filler contents (5–10%), suggesting a more efficient load transfer under compressive stress. Composites with up to 10% particulate presented a viable balance between mechanical performance and sustainability, showing potential for non-structural applications such as panels, coatings, and eco-friendly construction components. Overall, the results highlight the feasibility of using natural and mineral particulates as sustainable reinforcements, albeit with performance constraints at higher loadings.

## 1. Introduction

In the current context of climate change and increasing pressure for decarbonization, the development of sustainable polymer-based materials has become a strategic priority in materials science and engineering. In this scenario, polymer composites represent an important technological route, as they allow the combination of tailored mechanical performance with reduced material consumption and extended service life. Epoxy matrix composites, in particular, are widely used in structural and semi-structural applications due to their high strength, good adhesion, and chemical resistance, but their petrochemical origin and limited recyclability raise environmental concerns [1].

To mitigate these drawbacks, the partial replacement of conventional synthetic reinforcements by natural or waste-derived materials has been extensively investigated. Natural fibers and lignocellulosic fillers stand out due to their low density, renewability, wide availability, and lower environmental footprint, in addition to contributing to waste valorization when derived from post-consumer or industrial residues [2,3]. However, the incorporation of such materials into epoxy matrices often leads to challenges related to interfacial adhesion, moisture sensitivity, and variability in mechanical performance, which still limit their broader industrial adoption.

Another widely explored strategy to enhance the performance of sustainable epoxy composites is the incorporation of ceramic fillers, particularly layered silicates such as montmorillonite clay. Montmorillonite has attracted significant attention due to its high aspect ratio, large specific surface area, and ability to improve stiffness, thermal stability, and barrier properties when properly dispersed in polymer matrices [4,5,6]. Despite these advantages, clays are non-renewable materials, and their extraction is associated with environmental and social impacts, which must be balanced against their functional benefits in composite formulations [7].

Hybrid composites combining natural reinforcements and ceramic fillers have therefore emerged as a promising approach, aiming to synergistically combine sustainability with improved mechanical performance. Rather than relying on a single reinforcement mechanism, hybrid systems can exploit load transfer from the polymer matrix to both fibrous and particulate phases, potentially compensating for individual limitations. Previous studies have demonstrated that the mechanical response of hybrid epoxy composites is strongly dependent on filler type, particle size, dispersion quality, and processing route, particularly for impact and flexural loading conditions [8,9,10,11]. Nevertheless, inconsistencies in performance, agglomeration effects, and processing-related constraints remain critical challenges in this field.

In addition, most existing studies focus on virgin or purpose-grown natural fibers, while comparatively fewer investigations address the use of real construction or industrial waste as reinforcing phases, despite their high potential for large-scale waste reduction and circular economy implementation [12,13,14,15]. Construction-derived lignocellulosic residues, such as eucalyptus shoring waste, represent an underexplored resource with significant availability in civil engineering activities, especially in emerging economies [15,16,17].

Within this context, the present study aims to evaluate the mechanical behavior of epoxy-based composites incorporating eucalyptus powder obtained from post-consumer construction waste and montmorillonite clay as individual and hybrid reinforcements. The specific objectives are to (i) investigate the effect of different volume fractions (5%, 10%, and 20%) of eucalyptus powder and clay on the processing behavior of the epoxy resin; (ii) systematically assess and compare the impact, flexural, and compressive strength of single-phase and hybrid composite formulations; and (iii) correlate the microstructural features and dispersion state of the reinforcements with the observed mechanical performance and failure mechanisms. The practical relevance of this study lies in demonstrating the feasibility of valorizing construction waste as a reinforcing phase in polymer composites, contributing to waste reduction and the development of more sustainable materials.

Although a direct comparison with freshly obtained eucalyptus powder was not performed, the recycled material from civil construction shoring scraps in Campos dos Goytacazes was ground and sieved to ensure uniform particle size distribution. Potential differences in fiber quality due to prior exposure, aging, or surface contamination could, however, affect fiber-matrix consistency and adhesion, representing a limitation to be addressed in future analyses. The origin of the material (construction scraps) is a crucial factor in understanding these potential variations in quality and consistency.

## 2. Materials and Methods

**Figure 1:**
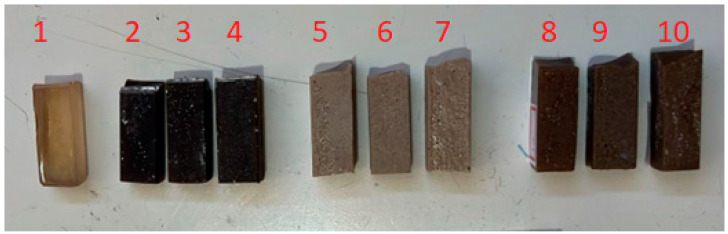
Impact specimens test. Legend can be found in Table 1.

The eucalyptus material was sourced from a civil construction company in Campos dos Goytacazes, Brazil, provided as post-consumer waste after being used as structural shoring in a masonry construction project. The material was received in its original log form (Figure 1). Subsequently, the shoring material was mechanically reduced into chips, which were then milled by knife and ball milling into powder. This powder was sieved through a 0.150 mm mesh to isolate the finest particulates for composite application.

Following sieving, the eucalyptus powder was dried in an oven at 60 °C for 24 h. The dried particulates were then mixed with a commercial bisphenol-A diglycidyl ether (DGEBA) epoxy resin and a diethylenetriamine (DETA) hardener, using a stoichiometric ratio corresponding to stoichiometric ratio of PHR = 16, provided by Sialox (Cherry Hill, NJ, USA) and supplied as Avipol Resin 2121 (São Paulo, Brazil) with Hardener 1050. The formulations investigated in this study are summarized in Table 1, which details the volumetric fractions (%) of epoxy resin, eucalyptus powder, and montmorillonite clay.

These formulations were defined with a maximum of 20% total volumetric fraction, considering viscosity, as adding more than 20% particulate makes the mixing process significantly more difficult and hinders its use for certain applications. It is important to note that no quantitative viscosity or rheological analyses were performed in this study. The reported increase in viscosity at particulate contents above 20% was qualitatively observed during the manual mixing process, where higher particulate fractions resulted in noticeable thickening and reduced flowability of the resin. Future studies including rheological evaluation could provide a more precise correlation between particulate loading and processability.

The montmorillonite clay used in this study was employed as natural form, without any chemical modification or surface treatment. The objective was to evaluate the intrinsic effect of raw clay incorporation on the mechanical performance of the composites [5,6]. Although treatments such as organophilization or silane functionalization are known to enhance interfacial adhesion and dispersion within the polymer matrix this work intentionally focused on the unmodified material to assess its feasibility as a low-cost and environmentally simpler alternative.

**Figure 2:**
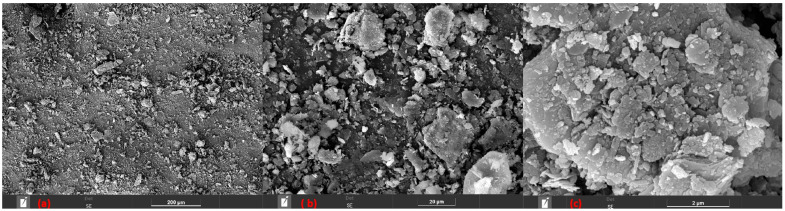
(**a**) Montmorillonite clay SEM at 400×; (**b**) montmorillonite clay SEM at 3 kx; (**c**) SEM of montmorillonite clay with 50 kx magnification.

The specimens can be seen in Figure 2, where the difference in shading is noticeable: the formulations with eucalyptus have a dark brown tone, those with clay have a beige tone, and the hybrid on es have a brown coloration.

### 2.1. SEM

To determine the morphology of the particulates used, scanning electron microscopy of field emisison gun (SEM-FEG) was performed using the Tescan Mira 4 (TESCAN, Brno, Czech Republic) equipment available at the Advanced Materials Laboratory (LAMAV) of the State University of Northern Rio de Janeiro (UENF). Zoom levels between 100× and 50,000× were applied.

### 2.2. Impact Strength

To measure the impact resistance, the Izod impact test was conducted in accordance with the ASTM D256 (2023) [18] standard, using a PANTEC XC-50 pendulum (PANTEC S.r.l., Turin, Italy) model with a 22J hammer available at LAMAV of UENF.

The analyzed samples will have approximate dimensions of 12.7 × 62.5 × 10 mm, featuring a “V”-shaped notch (45° angle) with a depth of 2.54 mm. At least five specimens of each formulation will be tested, resulting in a total of 50 impact specimens. Although the Izod impact test (ASTM D256) was used to assess impact resistance, it provides limited information about the fracture mechanics of the composites. Further evaluation through fracture toughness tests, such as single-edge notch bending (SENB) or compact tension (CT) methods, could offer deeper understanding of crack initiation and propagation mechanisms and their relation to fiber–matrix interfacial adhesion in future studies.

### 2.3. Flexural Strength

Six rectangular cross-section specimens were developed for each proposed formulation by three-point bending tests, with approximate dimensions of 6.0 × 1.5 × 1.5 cm, corresponding to a total of 60 flexural test specimens. The three-point bending tests were conducted using the Instron 5582 universal testing machine (Instron, Norwood, MA, USA) available at LAMAV of UENF, following the recommendations of the ASTM C580 [19] standard.

### 2.4. Compressive Strength

For this test, six specimens of each formulation were prepared, with approximate dimensions of 25.0 mm in diameter and 25.0 mm in height, in accordance with the ASTM C579 [20] standard, resulting in 60 compressive specimens analyzed in total. They were molded in an open silicone mold and cured at room temperature for 7 days.

Subsequently, the specimens were tested for compressive strength using the aforementioned Instron universal testing machine. The test was conducted at room temperature, with a speed of 1.3 mm/min and without shock.

## 3. Results and Discussion

### 3.1. SEM

#### 3.1.1. Montmorillonite Powder SEM

The present SEM micrographs of the montmorillonite clay powder at increasing magnifications, revealing important morphological characteristics that directly influence its dispersion behavior and mechanical performance when incorporated into the epoxy matrix. In Figure 2a, the powder exhibits a broad particle size distribution, with the coexistence of very fine particulates and larger grains. The particles predominantly display rounded to polygonal geometries, which are typical of mechanically processed clay minerals and suggest fracture-dominated size reduction rather than exfoliation.

In Figure 2b, it becomes evident that many of the larger particles are not monolithic grains but rather agglomerates composed of numerous finer plate-like particulates clustered together. This agglomeration behavior is characteristic of montmorillonite clays due to strong interparticle forces, such as van der Waals interactions and electrostatic attraction between stacked layers. The presence of these agglomerates indicates limited particle separation during processing, which may compromise homogeneous stress transfer within the composite and promote stress concentration sites under mechanical loading. In Figure 2c, the characteristic layered morphology of smectite clays is clearly observed. The lamellar structure, consisting of stacked silicate sheets, confirms the intrinsic plate-like nature of montmorillonite. Additionally, pores and voids of varying sizes are randomly distributed on the particle surfaces, likely resulting from interlayer spacing and mechanical fragmentation. While this porous and layered morphology can potentially enhance mechanical interlocking with the epoxy matrix under compressive loading, it may also facilitate resin entrapment and void formation if dispersion is inadequate.

#### 3.1.2. Eucalyptus Powder SEM

**Figure 3:**
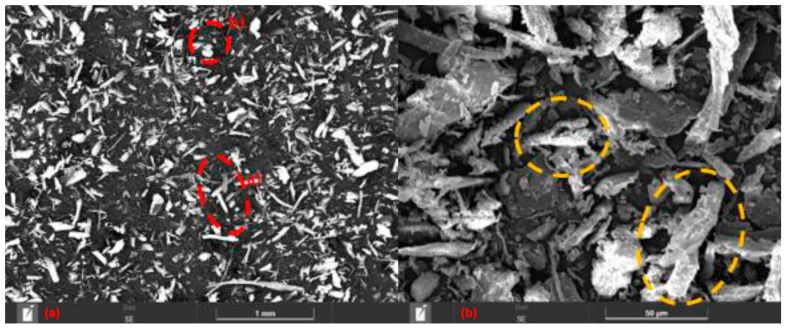
(**a**,**b**) SEM of eucalyptus powder with 100× magnification.

The microscopy of the eucalyptus shown in Figure 3a demonstrates that these particulates exhibit two distinct particulate morphologies, as in highlights a and b, dispersed in a heterogeneous and random manner. These morphologies consist of: (a), more elongated fibers, even after the grinding and sieving process, and (b), some grains with more rounded dimensions and closer to each other. It is also possible to observe, the presence of irregularities and fractured regions in the fiber (marked in yellow), resulting from the grinding and sieving process. Such characteristics tend to worsen the adhesion between the fiber and the matrix. Considering the irregular and torn morphology of the eucalyptus fibers, pretreatment methods such as alkali treatment or silane coupling could improve fiber integrity and adhesion to the epoxy matrix. As a result, the particulate may end up acting as a filler rather than a reinforcement within the composite, since being dispersed in the matrix at random angles, it can create points of weakness in the material [21].

In this study, the particulates were dispersed manually using mechanical stirring during the resin mixing process. No ultrasonication or chemical dispersing agents were employed. Although this method was effective for obtaining a visually homogeneous mixture, the use of advanced dispersion techniques such as ultrasonic treatment or high-shear mixing could further reduce agglomeration and improve particle distribution in future studies.

### 3.2. Impact Strength

**Figure 4:**
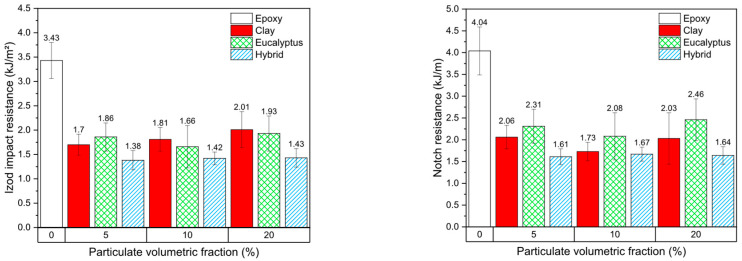
Izod impact strength and notch resistance results of the analyzed composites.

Figure 4 presents the results of Izod impact strength and notch resistance for the epoxy composites as a function of particulate volumetric fraction and reinforcement type. In contrast to the neat epoxy matrix, which exhibited the highest impact resistance, all composite formulations showed a marked reduction in both Izod impact strength and notch resistance, regardless of the type or amount of particulate reinforcement. Notably, no clear monotonic trend was observed with increasing volumetric fraction, indicating that the reduction in impact performance is primarily associated with the presence of particulates rather than their concentration alone.

For the Izod impact strength, values between 1.38 and 2.01 kJ·m^−2^ were obtained for the composite formulations, compared to 3.43 kJ·m^−2^ for the neat epoxy. Similarly, notch resistance values ranged from 1.51 to 2.46 kJ·m^−2^, whereas the pure epoxy reached 4.04 kJ·m^−2^. These results demonstrate that the incorporation of both eucalyptus powder and montmorillonite clay significantly reduced the energy absorption capacity of the epoxy matrix under impact loading. This behavior contrasts with some studies reported in the literature for epoxy composites reinforced with continuous or long natural fibers, which often exhibit improved or preserved impact resistance due to fiber pull-out and crack-bridging mechanisms [22,23]. In the present study, however, the eucalyptus reinforcement was introduced in particulate form, which limits energy dissipation mechanisms and promotes brittle fracture behavior. Similar observations were reported by Velasco et al. [24], who evaluated epoxy composites reinforced with coconut shell particles and highlighted the detrimental effect of particulate agglomeration on impact performance.

SEM observations revealed the presence of particulate agglomerates in the composites, particularly at higher volumetric fractions, such as 20%, where increased resin viscosity hindered effective mixing and dispersion. These agglomerated regions act as weak zones within the matrix, facilitating crack initiation and rapid propagation under dynamic loading conditions. Although no computational modeling or quantitative image analysis was employed to assess the degree of agglomeration, the qualitative evaluation of SEM micrographs clearly indicated heterogeneous particulate distribution.

Comparable trends were also reported by Velasco et al. [21] in epoxy composites reinforced with industrial gypsum particles, where a reduction in impact strength was attributed to poor interfacial adhesion and an increased presence of voids relative to the neat resin. In impact-dominated loading, such defects severely impair the ability of the material to dissipate energy, resulting in catastrophic failure with limited plastic deformation.

Overall, the reduction in impact resistance observed in all composite formulations relative to the neat epoxy matrix can be attributed to a combination of microstructural and interfacial factors, including:

(i) poor adhesion between the particulate reinforcement and the epoxy matrix [25];
(ii) void formation associated with processing and increased viscosity [21];
(iii) particulate agglomeration at higher volumetric fractions [23];
(iv) facilitated crack propagation due to the interconnection of voids and agglomerates [22]; and
(v) the predominance of particulates acting as inert fillers rather than effective reinforcements under impact loading [25].

### 3.3. Flexural Strength

**Figure 5:**
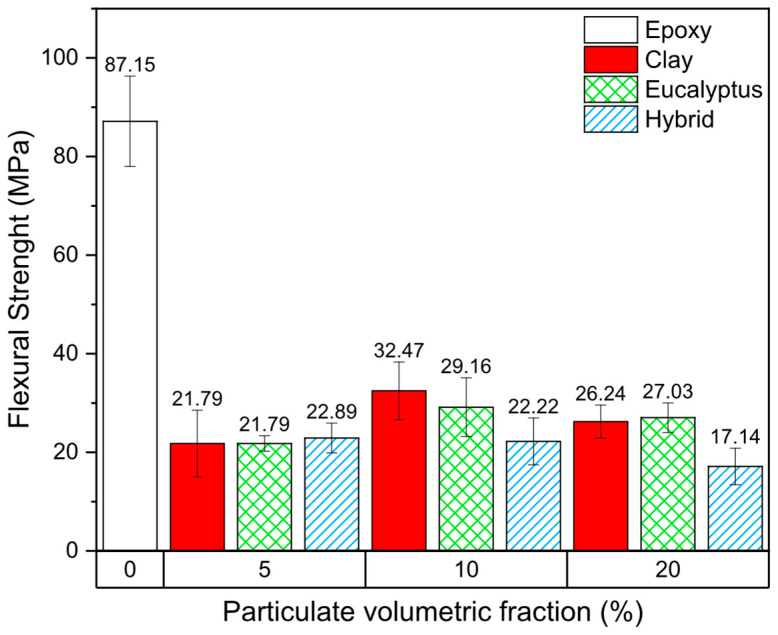
Flexural strength results of the analyzed composites.

Figure 5 presents the flexural strength results of the epoxy composites as a function of particulate volumetric fraction and reinforcement type. In contrast to the compressive behavior, all composite formulations exhibited a marked reduction in flexural strength when compared to the neat epoxy resin (87.15 MPa), indicating that the incorporation of particulate reinforcements had a detrimental effect under bending loads. This pronounced sensitivity to flexural loading highlights the different stress states involved, in which tensile stresses dominate on one side of the specimen and are strongly influenced by defects and interfacial discontinuities.

For the clay-reinforced composites, flexural strength values of 21.79 MPa, 32.47 MPa, and 26.24 MPa were obtained for volumetric fractions of 5%, 10%, and 20%, respectively. Although a slight increase was observed at 10% compared to 5%, all values remained substantially lower than that of the neat epoxy. This behavior suggests that, under flexural loading, the presence of clay particles was insufficient to promote effective stress transfer and instead contributed to premature crack initiation.

A similar trend was observed for the eucalyptus powder-reinforced composites. Flexural strength values of 21.79 MPa, 29.16 MPa, and 27.03 MPa were recorded for 5%, 10%, and 20% volumetric fractions, respectively. Despite minor variations among the formulations, none of the compositions approached the performance of the unfilled resin, indicating that the lignocellulosic particulates acted predominantly as stress concentrators under bending rather than as effective reinforcements.

The hybrid composites combining eucalyptus powder and montmorillonite clay exhibited flexural strength values of 22.89 MPa, 22.22 MPa, and 17.14 MPa for 5%, 10%, and 20% volumetric fractions, respectively. The progressive reduction observed with increasing filler content further confirms the absence of a synergistic reinforcement effect in flexural loading conditions. On the contrary, the coexistence of two particulate phases likely intensified heterogeneity and defect density, leading to earlier failure.

These results are consistent with those reported by Velasco et al. [24], who also observed a reduction in flexural strength for epoxy composites reinforced with chamotte powder, coconut shell powder, and hybrid systems. In that study, the authors demonstrated that specimens fabricated under vacuum exhibited significantly improved flexural performance, emphasizing the critical role of porosity control in particulate-filled epoxy composites.

Other studies focusing on hybrid epoxy composites reinforced with silica nanoparticles and jute fibers at low volumetric fractions (up to 4%) reported that flexural strength remained close to that of neat epoxy up to approximately 3%, followed by a sharp decrease at higher contents [26,27]. This comparison highlights that the high particulate loadings employed in the present study, combined with the use of micron-sized fillers, significantly increase the likelihood of defect formation and stress localization under bending.

Microstructural observations from SEM analyses revealed a non-uniform particle size distribution for both eucalyptus powder and montmorillonite clay. Larger particles are known to act as preferential sites for stress concentration, facilitating crack initiation, while finer particles may enhance stress transfer when well dispersed. The coexistence of these different particle scales, together with incomplete wetting of the particulates by the epoxy matrix, likely contributed to the reduced flexural performance observed across all formulations. From a mechanistic perspective, the severe reduction in flexural strength can be primarily attributed to increased porosity and weak interfacial bonding introduced by the particulate inclusions. Under bending loads, these defects facilitate crack propagation through the tensile region of the specimen, leading to premature failure. Consequently, while the studied composites demonstrated acceptable compressive performance and sustainability benefits, their flexural behavior remains a critical limitation that must be addressed through improved processing routes, such as vacuum-assisted molding, surface treatments, or optimized particle size distribution.

### 3.4. Compressive Strength

**Figure 6:**
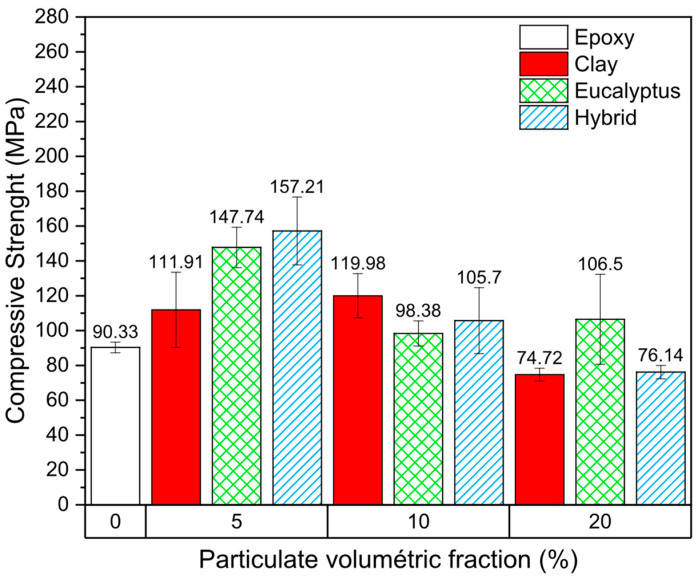
Compressive strength results of the analyzed composites.

Figure 6 presents the compressive strength results of the epoxy composites as a function of particulate volumetric fraction and reinforcement type. Overall, most composite formulations exhibited higher compressive strength than the neat epoxy resin (90.33 MPa), indicating that the incorporation of particulate reinforcements was generally effective in enhancing load-bearing capacity under compressive loading.

For the clay-reinforced composites, an increase in compressive strength was observed at volumetric fractions of 5% and 10%, with values of 111.91 MPa and 119.98 MPa, respectively. The relatively small difference between these two compositions suggests that, within this range, the clay particles contributed efficiently to stress transfer without significantly compromising matrix continuity. However, a pronounced reduction in compressive strength was observed at the 20% volumetric fraction (74.72 MPa), indicating the existence of a critical filler content beyond which the reinforcing effect is offset by microstructural defects.

Although post-failure microstructural analysis was not performed for these compositions, this behavior can be attributed to increased particle agglomeration and void formation at higher filler contents, which act as stress concentrators and facilitate crack initiation and propagation under compressive loads. This trend is consistent with the findings reported by Rocha Júnior et al. [28], who observed a similar reduction in compressive strength for epoxy composites reinforced with chamotte at a 20% volumetric fraction.

The composites reinforced with eucalyptus powder exhibited a non-monotonic response to increasing particulate content. At 5% and 20% volumetric fractions, the compressive strength increased significantly compared to the neat epoxy, reaching values of 147.74 MPa and 106.50 MPa, respectively. In contrast, the composite containing 10% eucalyptus powder showed no significant improvement (98.38 MPa), indicating a sensitivity of compressive performance to particulate distribution and interfacial quality. This behavior differs from that reported by Oliveira et al. [29], who observed a reduction in compressive strength starting at a 10% volumetric fraction in epoxy composites reinforced with eucalyptus powder, which was attributed to poor fiber–matrix adhesion and void formation. The discrepancy between these results highlights the strong influence of particle size distribution, surface condition, and processing route on the mechanical response of lignocellulosic-filled epoxy systems.

Similar trends have been reported for other lignocellulosic particulates. Velasco et al. [21], for instance, observed an increase in compressive strength in epoxy composites reinforced with coconut shell powder at 10% and 20% volumetric fractions, followed by a significant reduction at higher contents (30–40%). These results reinforce the existence of an optimal filler range, in which crack propagation is hindered by the presence of well-dispersed particles, while excessive filler contents lead to structural discontinuities. From a mechanistic standpoint, the improvement in compressive strength at lower volumetric fractions can be associated with the ability of rigid particulates to restrict matrix deformation and impede crack growth. Under compressive loading, well-dispersed particles act as physical barriers to crack propagation, enhancing resistance to localized failure. Conversely, at higher filler contents, particle–particle interactions and insufficient matrix wetting reduce the effectiveness of load transfer, resulting in premature failure.

Regarding the hybrid formulations combining eucalyptus powder and montmorillonite clay, no significant synergistic effect was observed. The compressive strength values did not exceed those of the corresponding single-particulate composites, suggesting limited interfacial compatibility between the two reinforcing phases and the epoxy matrix under the processing conditions employed. This absence of synergy indicates that, for compressive loading, the combined use of lignocellulosic and ceramic particulates does not necessarily translate into enhanced mechanical performance without further optimization of particle dispersion and interfacial bonding. Despite the observed reduction in compressive strength for some 20% volumetric fraction formulations, the measured values (74.72 MPa and 76.14 MPa) remain within an acceptable range for non-structural and semi-structural applications. From a sustainability perspective, formulations containing up to 10% particulate reinforcement represent a favorable balance between mechanical performance and environmental benefit, supporting the feasibility of using construction-derived eucalyptus waste as a reinforcing phase in epoxy composites.

The present study also investigated whether a combination of eucalyptus powder and montmorillonite clay could generate a similar synergistic effect. However, the results revealed that hybrid formulations did not exhibit significant improvement over single-particulate composites, suggesting limited interfacial compatibility and absence of synergistic reinforcement behavior under the tested conditions.

### 3.5. Sustainability Aspects of Reinforcements

In the pursuit of sustainable composite materials, the main sustainability aspects of the reinforcing phases used in this study can be highlighted as waste valorization, renewability, reduced environmental burden during production, and potential service-life extension, namely for eucalyptus powder and montmorillonite clay. The eucalyptus powder is a recycled lignocellulosic residue derived from building construction activities, which directly promotes waste valorization and reduces the demand for virgin raw materials, in accordance with circular economy principles [30,31,32]. As a renewable biomass-based material, eucalyptus fibers generally present a lower environmental footprint when compared to conventional synthetic reinforcements.

Although a comprehensive Life Cycle Assessment (LCA) is beyond the scope of this work, a qualitative sustainability assessment based on established LCA principles is provided to contextualize the environmental benefits of the reinforcements. During the production stage, the use of eucalyptus powder sourced from construction waste significantly reduces environmental burdens by avoiding additional raw material extraction and minimizing landfill disposal impacts [33,34]. This upcycling strategy typically leads to lower energy consumption and reduced greenhouse gas emissions when compared to composites reinforced with virgin synthetic fibers. Regarding the mineral reinforcement, montmorillonite clay does require extraction and processing; however, its environmental impact per unit mass is relatively low due to its natural abundance and limited refinement requirements [35,36]. Moreover, its incorporation at low volumetric fractions allows relevant improvements in mechanical and barrier properties, increasing material efficiency without substantially increasing environmental load.

During the use phase, both reinforcements may contribute to extending the service life of epoxy-based composites by enhancing specific mechanical properties, which can reduce maintenance needs and delay replacement. At the end-of-life stage, the epoxy matrix remains the main environmental limitation due to its non-biodegradable nature and restricted recyclability [37,38]. Nevertheless, the incorporation of recycled lignocellulosic particulates improves the overall sustainability profile of the composite, resulting in a lower carbon footprint compared to fully synthetic reinforcement systems. Additionally, the use of montmorillonite clay is justified by its ability to enhance composite performance at low filler contents, indirectly supporting sustainability through extended product lifespan [38,39,40,41,42].

## 4. Conclusions

This study demonstrated that the incorporation of eucalyptus powder and montmorillonite clay significantly influences the processing and mechanical behavior of epoxy composites. High particulate contents (≥20%) increased resin viscosity, hindering processing and specimen fabrication. All composite formulations exhibited reduced impact and flexural strength compared to neat epoxy, mainly due to poor interfacial adhesion, particle agglomeration, and void formation, causing the reinforcements to behave primarily as fillers. In contrast, composites containing up to 10% particulate showed improved compressive strength, attributed to more efficient load transfer and the ability of rigid particles to restrict matrix deformation and hinder crack propagation. Considering their sustainable origin and mechanical response, these composites show potential for non-structural applications, particularly as anticorrosive and anti-erosive coatings.

Future work could explore the use of coupling agents or surface treatments to improve interfacial adhesion between the matrix and the particulates. For instance, silane-based compatibilizers or modified epoxy systems containing reactive diluents might enhance bonding with lignocellulosic fillers and clay, leading to improved mechanical performance.

## Figures and Tables

**Table 1:** Compositions.

No	Reference	Composition (%)		
		Epoxy	Eucalyptus	Montmorillonite
1	RE+0	100	-	-
2	RE+EU5%	95	5	-
3	RE+EU10%	90	10	-
4	RE+EU20%	80	20	
5	RE+AR5%	95	-	5
6	RE+AR10%	90	-	10
7	RE+AR20%	80	-	20
8	RE+EU2.5%+AR2.5%	95	2.5	2.5
9	RE+EU5%+AR5%	90	5	5
10	RE+EU10%+AR10%	80	10	10

## Data Availability

The original contributions presented in this study are included in the article. Further inquiries can be directed to the corresponding author.

## Abbreviations

The following abbreviations are used in this manuscript:

UENF	State University of the North of Rio de Janeiro Darcy Ribeiro
LAMAV	Advanced Materials Laboratory
SEM	Scanning Electronic Microscopy
